
JOURNAL INFORMATION
==============================
NLM Title Abbreviation: Atlanta Med Surg J (1884)
NLM Journal ID: 101251623

Atlanta Medical and Surgical Journal (1884)

Publisher: Biomedical Journal Digitization Project

ARTICLE INFORMATION
==============================
PMCID: PMC9199304
Article version: 1
Subjects: Cover
Print publication date: 1898 Dec
Volume: 15
Volume Series: New Ser
Issue: 10
License: This work is free of known copyright restrictions. For more information, please see PMC Back Issue Digitization.
License URL: https://creativecommons.org/publicdomain/mark/1.0/

==============================

PHILLIPS' PHOSPHO-MURIATE OF QUININE, COMP.
(the Soluble Phosphates, with Muriate of Quinine, Iron and Strychnia)
IN DEFICIENCY OF THE PHOSPHATES, LACK OF NERVE TONE, MALARIAL MANIFESTATIONS, CONVALESCENCE FROM EXANTHEMATA, ETC.—WILL NEVER DISAPPOINT.
BEWARE OF THE MANY IMITATIONS. PRESCRIBE "PHILLIPS'".
THE CHAS. H. PHILLIPS CHEMICAL CO., 77 Pine Street, NEW YORK.
Please mention this Journal.

LIBRARY
SURGEON GENERAL'S OFFICE
DEC.-10-1898

ESTABLISHED 1855. SUBSCRIPTION, $2.00.

### ATLANTA MEDICAL AND SURGICAL JOURNAL.

VOLUME XV NEW SERIES. Atlanta, Ga., December, 1898. No. 10.

ARSENAURO
AND
MERCAURO,
THE BEST KNOWN
MAKERS AND BUILDERS
OF
BLOOD
Please mention this Journal.

Published by THE FRANKLIN PRINTING AND PUBLISHING CO., Atlanta, Ga.
Entered at the Post-Office at Atlanta, Ga., as second-class matter.
